# Multiscale Simulations Suggest a Mechanism for the Association of the Dok7 PH Domain with PIP-Containing Membranes

**DOI:** 10.1371/journal.pcbi.1005028

**Published:** 2016-07-26

**Authors:** Amanda Buyan, Antreas C. Kalli, Mark S. P. Sansom

**Affiliations:** Department of Biochemistry, University of Oxford, Oxford, United Kingdom; University of Houston, UNITED STATES

## Abstract

Dok7 is a peripheral membrane protein that is associated with the MuSK receptor tyrosine kinase. Formation of the Dok7/MuSK/membrane complex is required for the activation of MuSK. This is a key step in the complex exchange of signals between neuron and muscle, which lead to neuromuscular junction formation, dysfunction of which is associated with congenital myasthenic syndromes. The Dok7 structure consists of a Pleckstrin Homology (PH) domain and a Phosphotyrosine Binding (PTB) domain. The mechanism of the Dok7 association with the membrane remains largely unknown. Using multi-scale molecular dynamics simulations we have explored the formation of the Dok7 PH/membrane complex. Our simulations indicate that the PH domain of Dok7 associates with membranes containing phosphatidylinositol phosphates (PIPs) via interactions of the β1/β2, β3/β4, and β5/β6 loops, which together form a positively charged surface on the PH domain and interact with the negatively charged headgroups of PIP molecules. The initial encounter of the Dok7 PH domain is followed by formation of additional interactions with the lipid bilayer, and especially with PIP molecules, which stabilizes the Dok7 PH/membrane complex. We have quantified the binding of the PH domain to the model bilayers by calculating a density landscape for protein/membrane interactions. Detailed analysis of the PH/PIP interactions reveal both a canonical and an atypical site to be occupied by the anionic lipid. PH domain binding leads to local clustering of PIP molecules in the bilayer. Association of the Dok7 PH domain with PIP lipids is therefore seen as a key step in localization of Dok7 to the membrane and formation of a complex with MuSK.

## Author Summary

Neuromuscular junction formation and maintenance is an essential biological process, the disruption of which leads to congenital myasthenic syndromes and premature death. Dok7 is a key member in formation, maintenance and signaling in neuromuscular junctions. Dok7 is a peripheral membrane protein that is necessary for full activation of the receptor tyrosine kinase MuSK, a receptor tyrosine kinase residing in the postsynaptic membrane. The structure of Dok7 consists of both a PH domain and a PTB domain. The interaction of Dok7 with cell membranes is not well understood. Here, we use molecular simulations to show that the Dok7 PH domain preferentially binds to PIP lipid molecules when associating with a membrane. Dok7 interacts with the bilayer in both a *canonical* binding mode and an *alternative* binding mode. Our simulations also demonstrate the presence of both a canonical and an atypical binding site for PIPs in agreement with recent crystallographic studies of the ASAP1 PH domain. Analysis of density landscapes for the interaction of the Dok7 PH domain with PIP-containing lipid bilayers is also able to identify both *canonical* and *alternative* binding modes. Our improved understanding of how Dok7 interacts with a membrane is key to examining Dok7/MuSK signaling.

## Introduction

**Fig 1:**
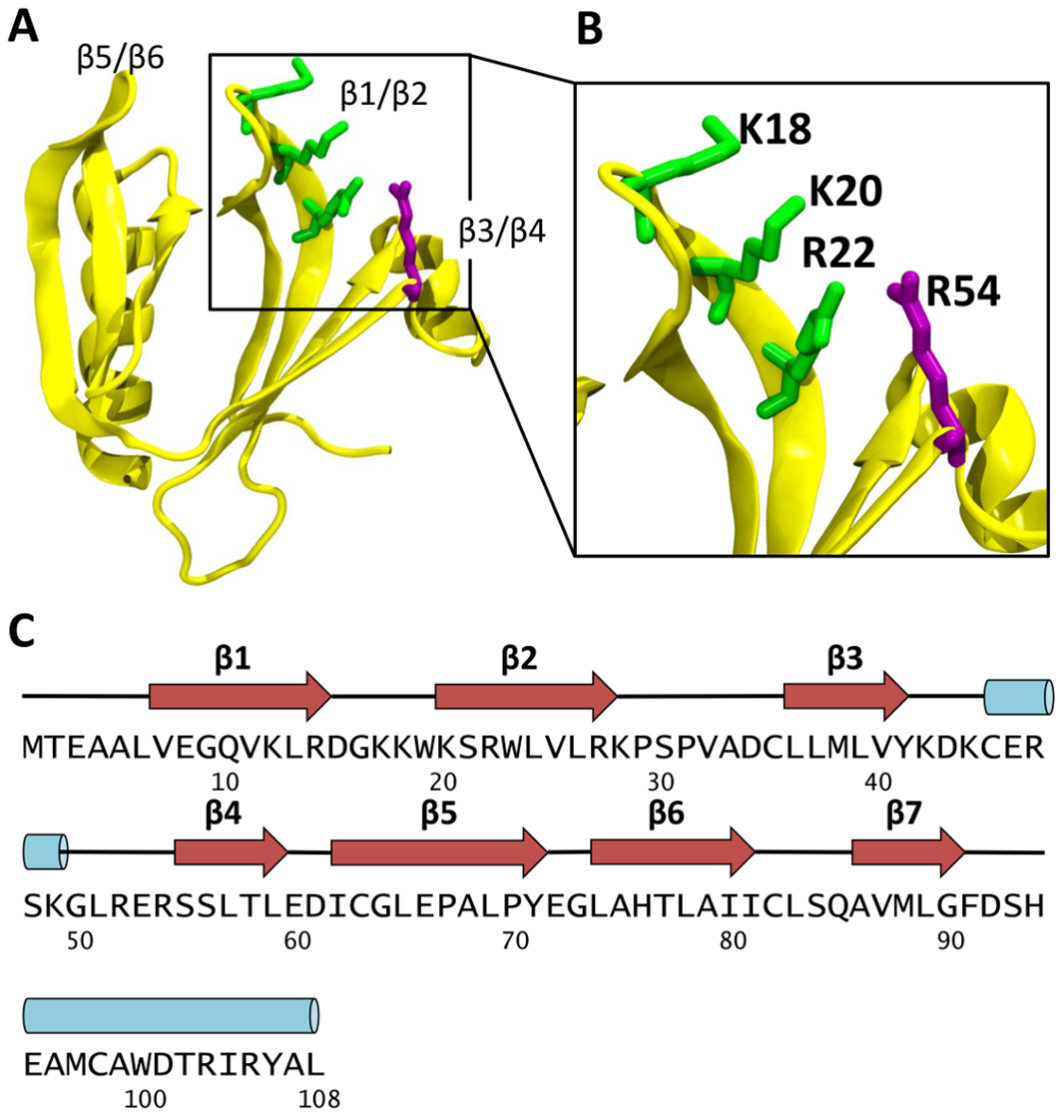
Previous structural studies of Dok7. **(A)** Structure of the PH Domain of Dok7 (PDB id: 3ML4), shown in yellow cartoon representation. **(B)** The four residues thought to be involved in PIP binding (K18, K20, R22 and R54) are in green or purple licorice representation. **(C)** The sequence of the Dok7 PH domain is shown, with the secondary structure indicated above the sequence. Black lines represent unstructured regions, red arrows represent β sheets, and blue cylinders represent α-helices.

Dok7 (Downstream-of-Kinase 7) is a peripheral membrane protein and a member of the Dok family of proteins [1]. Dok7 is the cytoplasmic activator of muscle-specific kinase (MuSK) [2], which is involved in neuromuscular junction formation and maintenance [3–5]. Mutations in Dok7 lead to the disease *myasthenia gravis* [6–12], indicating that Dok7 plays a key role in the activation of MuSK. Dok7 contains a Pleckstrin Homology (PH) and a Phosphotyrosine Binding (PTB) domain [2], as seen in the structure: a dimer of the first 210 residues of Dok7 was co-crystallized with a phosphotyrosine-containing 13-mer peptide corresponding to the Dok7 binding site of MuSK [13]. Given that Dok7 contains a PH domain, it is likely that Dok7 may localize to cell membrane via lipid-mediated interactions (Fig 1A and 1B), but the mechanistic details of how the formation and stability of the Dok7 PH/membrane complex are as yet unknown.

PH domains are a structurally conserved family which have been shown to associate with phosphatidylinositol phosphates (PIPs; [14,15]). PH/PIP interactions help to localize PH domains to specific cellular membranes. Dok7 may interact with a number of PIP species [13], showing low micromolar affinities for (fluorescent derivatives of) PI(3,4,5)P_3_, PI(4,5P)_2_ and PI(3,4)P_2_ [13]. Mutation of a positive residue (R54A) on the β3/β4 loop of the Dok7 PH domain (Fig 1B and 1C) results in a >5-fold loss of PIP binding. R54 forms part of a cluster of basic residues (with K18, K20 and R22), which form a positively charged patch on the PH domain between the unstructured regions of β1/β2 and β3/β4 strands (Fig 1C), corresponding to the *canonical* binding site for PIPs in a number of PH domains [16,17]. The PTB domain of Dok7 binds to a phosphorylated tyrosine (pTyr553) in the juxtamembrane region of MuSK, enabling downstream signalling within the cell [18,19]. It has been shown that the juxtamembrane regions of all human receptor tyrosine kinases (including MuSK) are able to interact with PIPs [20]. Thus, PIP-mediated interactions of the PH domain would be expected to aid in localization of Dok7 close to the juxtamembrane region of MuSK.

Understanding the mechanism of association of the Dok7 PH domain with a model cell membrane will provide insights into the interactions stabilizing the Dok7/MuSK/membrane complex, since the PH orientation relative to the membrane is likely to play a key role in the Dok7/MuSK interaction. It has been shown that molecular dynamics (MD) simulations provide a tool for understanding the mechanism of association of peripheral proteins with models of cell membranes [21–23], having been applied to the membrane binding interactions of e.g. PTEN [24], auxilin [25], talin [26], NRas [27,28], and the canonical PH domain of GRP1 [29]. These simulation studies have revealed specific interactions of these proteins with lipid molecules, which are in good agreement with available experimental data.

In the current study, we employ a multi-scale MD simulation approach [21] to explore how the PH domain of Dok7 binds to model lipid bilayers with differing phospholipid and PIP contents. Our analysis employing the evaluation of interaction density landscapes is able to differentiate between *canonical* and *alternative* binding modes of the PH domain. Our results allow us to suggest a mechanism for PIP-mediated association of the PH domain of Dok7 with cell membranes.

## Results

### PH domain association with PIP-containing lipid bilayers

We first explored the association of the PH domain of Dok7 with a PIP-containing bilayer. In these simulations, the Dok7 PH domain was initially positioned ~7 nm away from a preformed lipid bilayer composed of 75% PC + 20% PS + 5% PIP_2_. An ensemble of 20 simulations each of duration 1 μs (see Table 1) was then performed.

**Table 1:** Summary of the simulations.

Simulation	Lipid Bilayer	Duration
CG-PH-pip2	PC/PS/PIP_2_ (75%/20%/5%)	20 x 1 μs
CG-PH-pip3	PC/PS/PIP_3_ (75%/20%/5%)	20 x 1 μs
CG-PH-pc	PC (100%)	20 x 1 μs
CG-PH-pcps	PC/PS (80%/20%)	20 x 1 μs
AT-PH-pip2	PC/PS/PIP_2_ (75%/20%/5%)	3 x 0.3 μs
AT-PH-pip3	PC/PS/PIP_3_ (75%/20%/5%)	3 x 0.3 μs

Summary of the simulations used in this study. The PIP_2_ simulations used PI(4,5)P_2_ (S1A Fig), and the PIP_3_ simulations used PI(3,4,5)P_3_ (S1B Fig).

**Fig 2:**
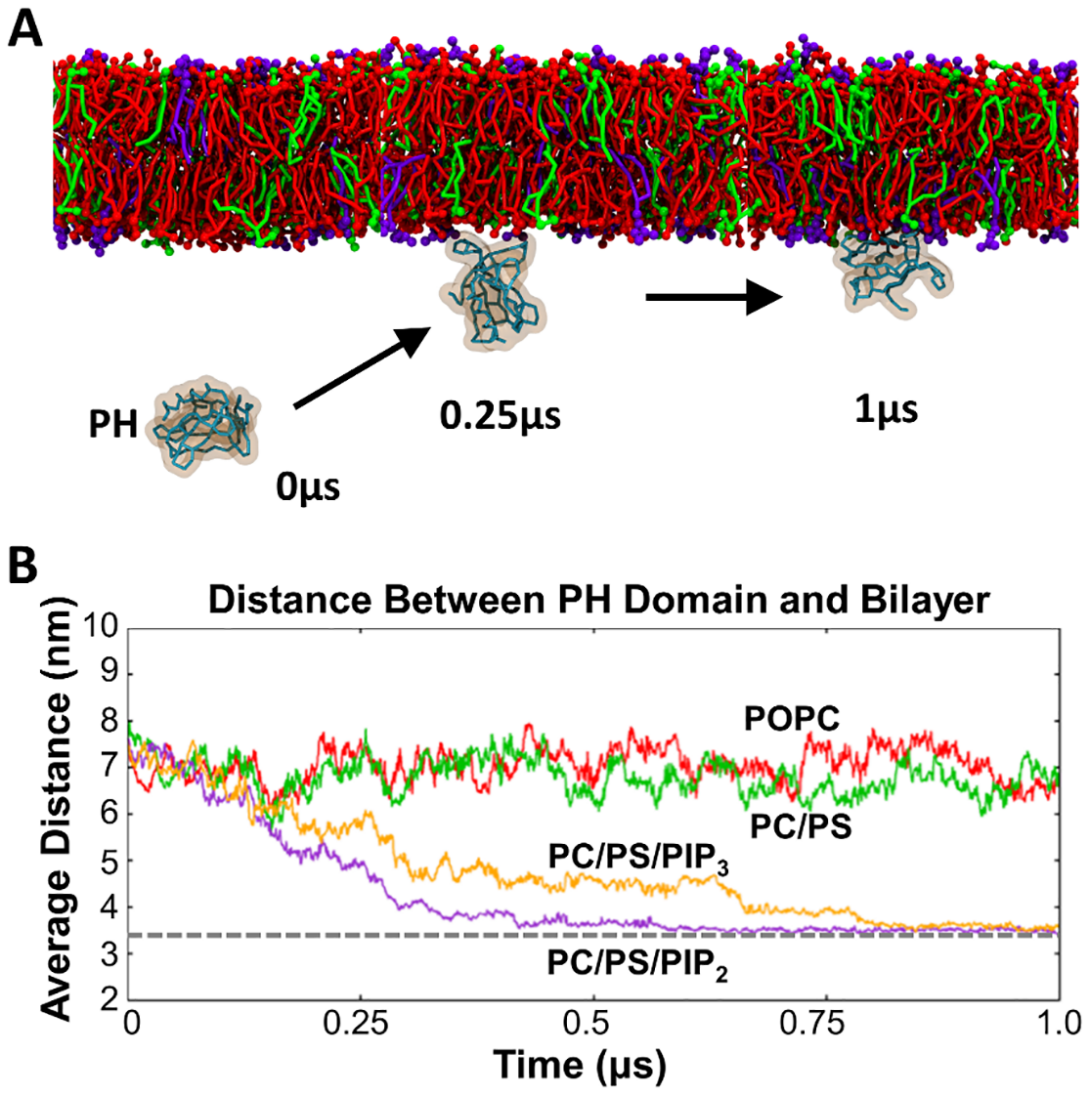
Simulation setup and bilayer composition dependence on binding. **(A)** Simulation set-up and progression. These are snapshots from a CG-PH-pip2 simulation (see Table 1 for details) at 0, 0.25 and 1 μs. PC molecules are shown in red, PS in green and PIP_2_ molecules in purple. The PH domain of Dok7 is shown as a cyan Cα trace with orange surface. **(B)** Average distances (from the 20 simulations in each ensemble) between the centre of mass of the PH domain and that of the bilayer as a function of time are shown for four different bilayers (PC, red; PC/PS, green; PC/PS/PIP_2_, purple; PC/PS/PIP_3_, orange) used in the PH domain simulations. The dotted grey horizontal line indicates the approximate position of a PH domain bound to the surface of the membrane.

During each simulation, the protein diffused in the aqueous solution before interacting with the bilayer (Fig 2A). In the majority of the individual simulations within the ensembles in which PIP_2_ molecules were present, the Dok7 PH domain associated with the bilayer surface within the first ~0.25 μs and remained bound for the remainder of the simulation (Fig 2B) without any dissociation events occurring. Simulations with a bilayer containing PIP_3_ yielded similar results, although it took on average a little longer (~0.75 μs, see Fig 2B) for the PH domain to bind to the bilayer, despite the greater negative charge on PIP_3_ compared to PIP_2_. Recent experimental studies yielded dissociation constants of Dok7 (PH-PTB) for (derivatized) PIP_2_ and PIP_3_ of 4 and 2 μM respectively [13]. In contrast, if either a zwitterionic bilayer (100% PC) or an anionic bilayer lacking PIP (80% PC + 20% PS) is used then no association of the Dok7 PH domain with the membrane is observed (Fig 2B), in good agreement with previous studies of other PH domains [30,31]. The sample size of 20 simulations per system is smaller than those explored in e.g. recent studies of TM helix dimerization [32,33]. However, convergence analysis (S2 Fig) suggested that the density landscapes were adequately sampled close to the bilayer by 20 simulations and indeed that even with small (~5) groups of simulations the density landscapes still resemble the overall landscape, with M1 as the predominant binding mode.

### Density landscapes

To further analyse the orientation and interactions of the Dok7 PH domain relative to different lipid bilayers, we have constructed two-dimensional density landscapes of the *R*_*zz*_ component of the rotational matrix of the PH domain (*R*_*zz*_; calculated using the *g_rotmat* command in GROMACS; see Methods), and of the distance of the PH domain from the bilayer (*d*_*z*_). In particular, *R*_*zz*_ is the *zz* component of the rotational matrix required for least squares fitting of a conformation onto a reference conformation, and *d*_*z*_ represents the perpendicular distance between the centres of mass of the PH domain and of the lipid bilayer. *R*_*zz*_ may adopt values from -1 to +1, and is equal to +1 when we use the preferred orientation as a reference conformation for the calculation of the rotational matrix. We note that related methodologies have been used to calculate the energy landscapes e.g. of cholesterol flip-flop [34] and of the insertion of hydrophobic peptides into model membranes [35].

**Fig 3:**
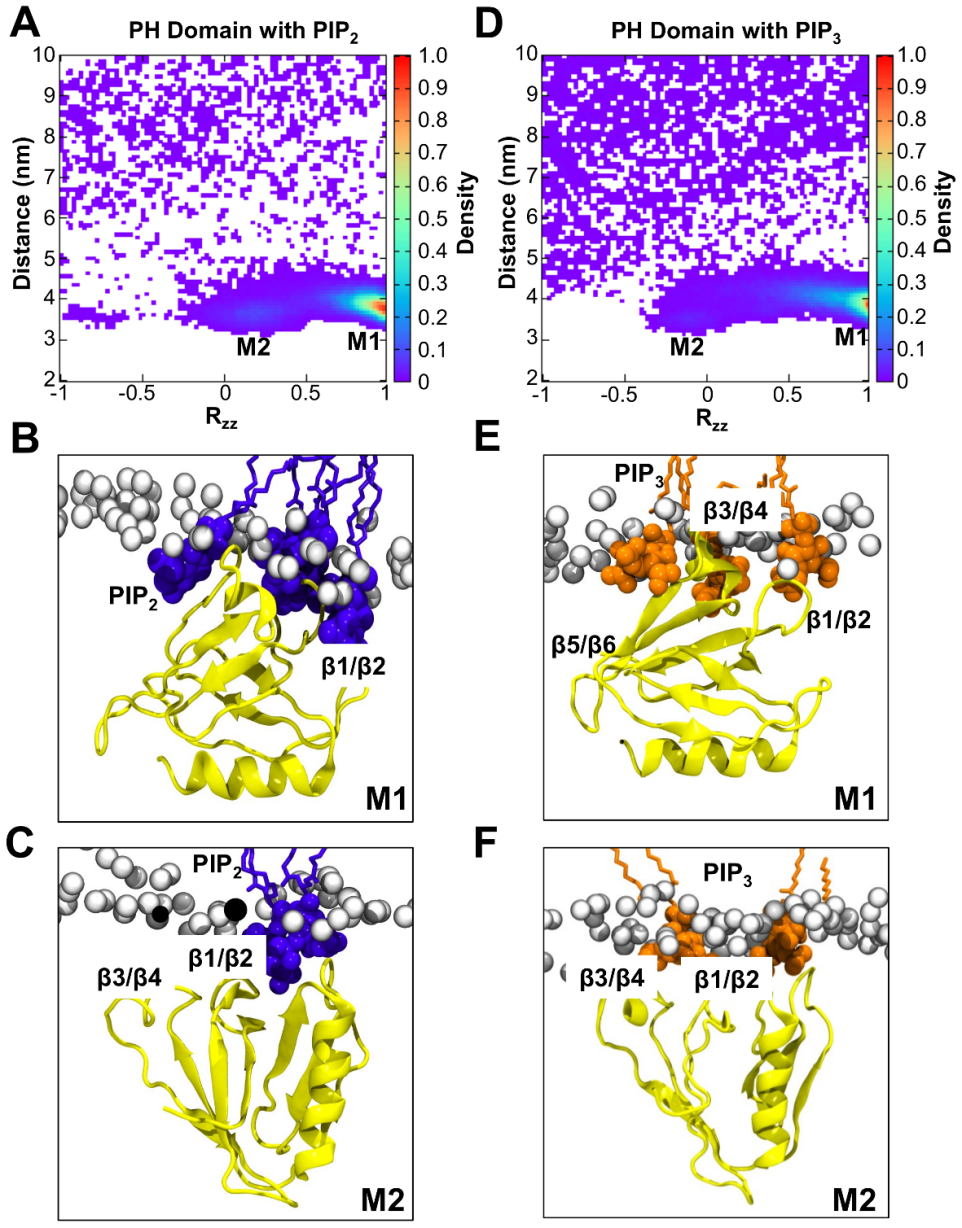
Density landscapes of the PH domain binding to PIP lipids. Densities of the PH domain binding to PIP_2_ **(A)** and PIP_3_ **(D)**. The density is shown as a function of domain orientation R_zz_ and the distance between the centres of mass of the protein and the bilayer. Representative structures of the M1 orientation with PIP molecules in both the A and C sites (see main text for details), and PIP molecules that are clustered around the protein, are shown in **(B)** for PIP_2_ and **(E)** for PIP_3_, whilst representative structures of M2 are shown in **(C)** for PIP_2_ and **(F)** for PIP_3_. Note that prior to the calculation the rotation of the protein in the *xy* plane was fitted using the *trjconv* command in GROMACS [37].

Analysis of the orientation of the Dok7 PH domain relative to a PIP_2_-containing membrane reveals that there is a clearly preferred binding mode of the PH domain to the bilayer (M1 in Fig 3A). In the preferred orientation, two regions of the PH domain interact with PIP_2_: the β1/β2 loop, which contains three cationic residues (K18, K20 and R22); and the β3/β4 loop (residues 42–54), which contains a short α-helix region at the C-terminus of which is R54 (Fig 3B). A similar preferred orientation was also observed in the density landscape for bilayers containing PIP_3_ (M1 in Fig 3D). The alternative orientation corresponds to the minor minimum in the PIP_2_-simulation landscape (R_zz_ = 0.2; M2 in Fig 3A—pictured in 3C for PIP_2_, and 3F for PIP_3_). In this orientation, the long α-helix (residues 94 to 107) of the PH domain is at an angle of ~60° relative to the plane of the bilayer, with β1/β2 and β3/β4 loops still maintaining contact with the bilayer. The M1 binding mode seen is very similar to a recently solved crystal structure of Arf GAP ASAP1 [36]. In the ASAP1 crystal structure (PBB id: 5C79), there are *two* anionic lipid binding sites: a *canonical* C-site between the β1/β2 and β3/β4 loops, and a second *atypical* A-site on the opposite face of the β1/β2 loops, towards β5/β6.

**Fig 4:**
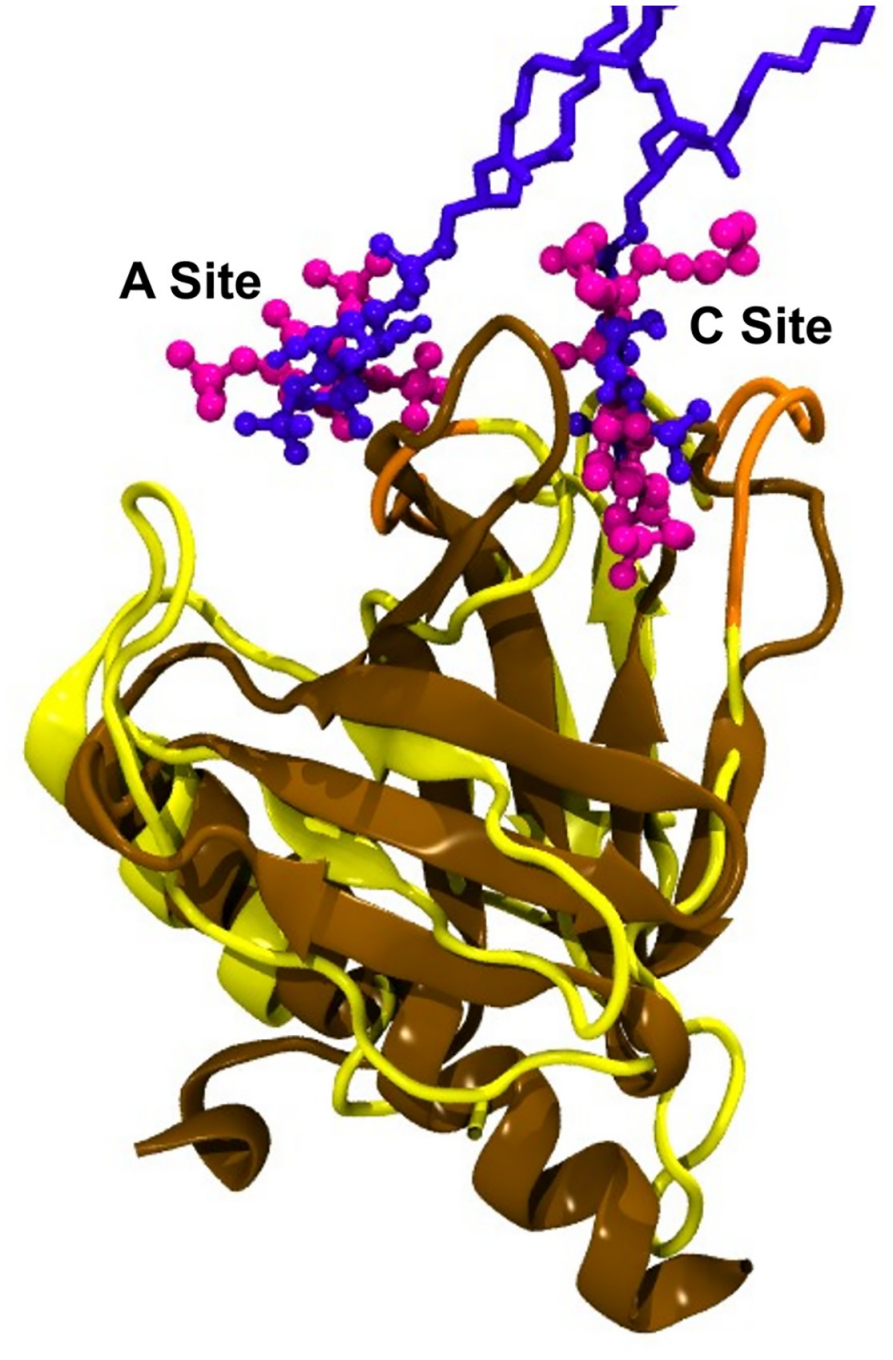
Comparison between the Arf GAP ASAP1 and Dok7 PH domains. Alignment of the Arf GAP ASAP1 (pdb id 5C79) with a representative structure from the simulation of the Dok7 PH domain in the presence of PIP_2_. The Dok7 PH domain is in yellow cartoon, and the PIP_2_ lipids from the simulation are in purple licorice and CPK. The Arf GAP ASAP1 backbone is in ochre cartoon, and the dibutyryl PIP_2_ molecules in the crystal structure are in CPK magenta.

The presence of two PIP-binding sites in the ASAP1 crystal structure is very similar to the arrangement of bound PIP_2_ molecules we see in the Dok7 simulations (Fig 4). Superimposition of the Dok7 and ASAP1 PH domains results in overlap of the headgroups of the PIP_2_ molecules at both the C and A sites. We note in passing that in the ASAP1 crystal structure the tails of the bound dibutyryl PIP_2_ would be pointing away from the bilayer.

To further quantify the similarities between the interactions of the Dok7 PH and ASAP1 PH domain with PIPs, 0.3 μs atomistic simulations of the Dok7 PH domain (see below) were compared to 0.5 μs simulations of the ASAP1 PH domain (the latter are described in detail in [38]). Lipid interactions for Dok7 alone (S3A Fig), ASAP1 alone (S3B Fig), and both aligned to each other (S3C Fig) show that the lipid interactions for both PH domains are similar. This observation is strengthened by quantification of the average number of contacts for both ASAP1 (S3D Fig) and Dok7 (S3E Fig). Both PH domains interact with lipids via their β1/β2 loop, as well as their β3/β4 loops, and lipids are present in both the *canonical* site, as well as the *atypical* site, in good agreement with experiments.

**Fig 5:**
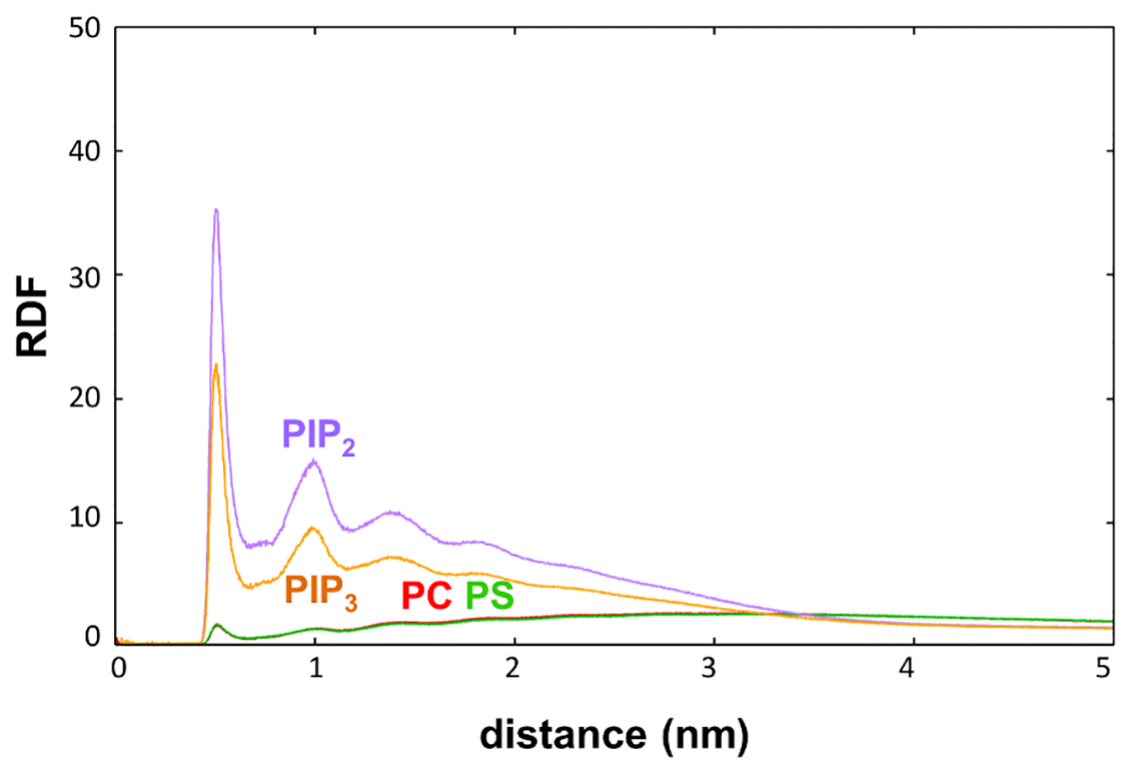
Average radial distribution function of PIP_2_ and PIP_3_ around Dok7’s PH domain. The average, over the 20 repeat simulations, radial distribution function of the coarse-grained simulations with PIP lipids. Averages for the PC/PS lipids were taken from the PC/PS/PIP_2_ simulations, whilst the PIP_3_ line is taken from the PC/PS/PIP_3_ simulations. The PC line is red, PS in green, PIP_2_ in purple, and PIP_3_ in orange.

**Fig 6:**
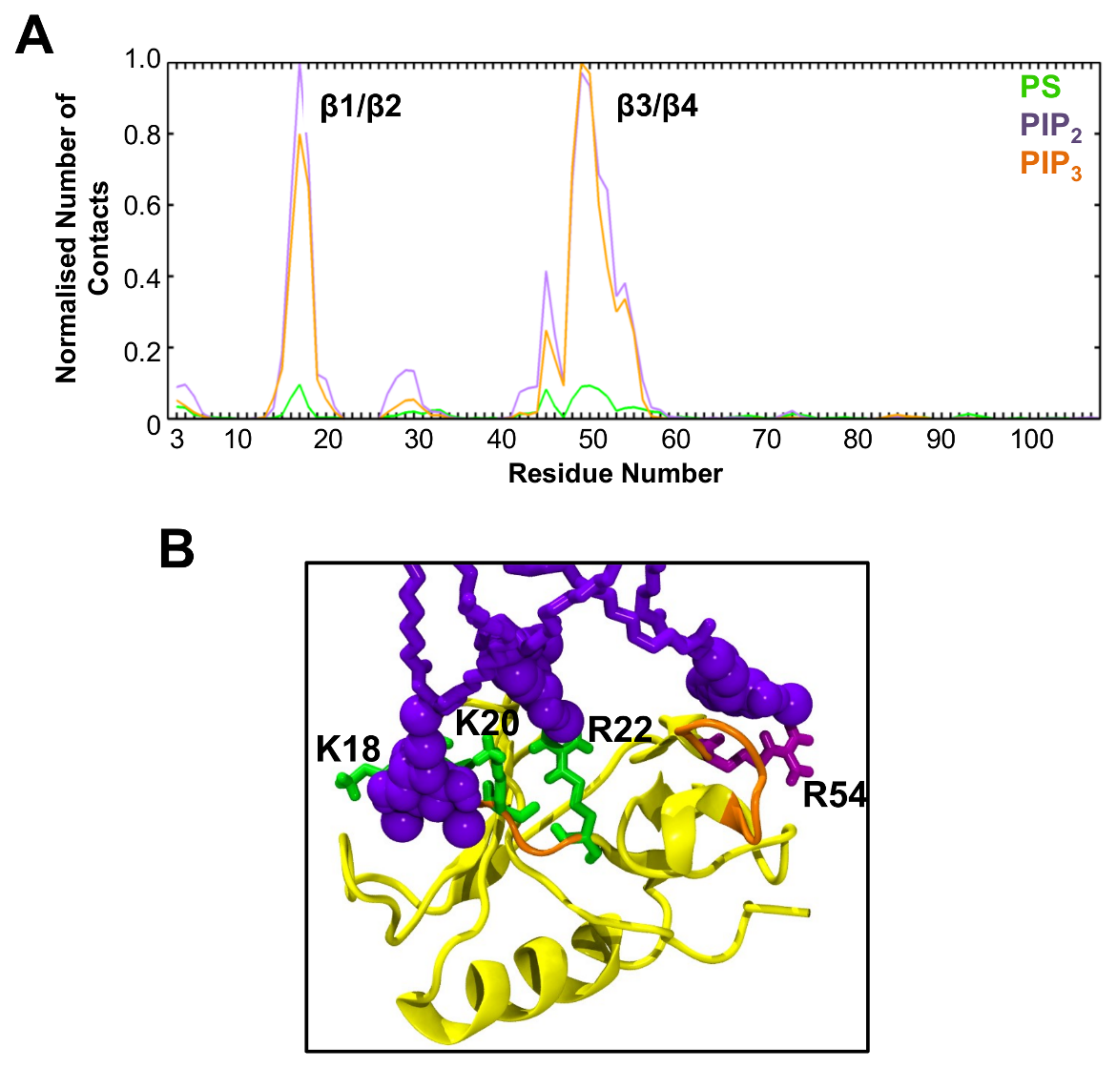
Lipid interactions between Dok7 and PIP_2_/PIP_3_. **(A)** Normalized interactions with PS, PIP_2_ and PIP_3_ as a function of residue number, derived from coarse-grained simulations. **(B)** Representative atomistic structure of the Dok7 PH domain interacting with PIPs in the bilayer. The backbone of the residues interacting with PIPs is highlighted in orange, and the main protein is in yellow cartoon. The residues proposed to interact with PIPs are shown in either green or purple licorice.

Evaluation of radial distribution functions (RDFs) of the different lipid species around the bound Dok7 PH domain (Fig 5) showed a large peak at 0.5 nm for PIP_2_ and PIP_3_, and a second peak at 1.0 nm, indicating that both PIP species preferentially cluster around the PH domain. Such clustering was not observed for PC or PS. Clustering of PIP lipids was observed only in the leaflet to which the protein was bound. Visual inspection of the simulation trajectories reveals that there are multiple PIP molecules surrounding the PH domain once it is bound to the bilayer, e.g. Fig 3B, 3C, 3E and 3F. As discussed above, the PIP lipids interact primarily with the basic residues of the β1/β2 loop and the small helix in the β3/β4, which contains R54 (see eg. Fig 6B). The main driving forces for these interactions appear to be the electrostatic interactions between the protein and the headgroups of the lipids (S4 Fig). In particular, from our analysis it seems that the protein interacts mainly with the bulky negatively charged PIP headgroups.

### Atomistic simulations

The preferred (i.e. most frequently observed) orientations of the PH domain when bound to either PIP_2_ or PIP_3_ as identified from the CG simulations were converted to atomistic representations and multiple (3 x 0.3 μs) atomistic simulations (Table 1) were performed. During the atomistic simulations, the PH domain retained its preferred orientation relative to the bilayer, its secondary structure (S5 Fig), and the main contacts identified from the CG-MD simulations (i.e. residues K18, K20, R22, and R54 interacting with PIP_2_; Fig 6A and 6B). For both PIP_2_ and PIP_3_, in some of the simulations the number of hydrogen bonds decreased at the first ~50 ns of simulations, although PIP_2_ maintained overall more hydrogen bonds than did PIP_3_ (S6 Fig). Examination of the interactions at the C-site (K18, K20, R22 and R54) showed that all residues maintained approximately 1–2 hydrogen bonds, to PIP_2_ (S7A Fig). Significantly, this was not the case for PIP_3_, which did not maintain most hydrogen bonds at the C site over the course of the 0.3 μs simulations (S7B Fig). This suggests that Dok7 forms a more stable complex with PIP_2_ than PIP_3_. This is further confirmed by breaking down the interactions between the headgroups, phosphates, and tails for the atomistic simulations (S8 Fig). In this analysis, it is shown that the headgroups are responsible for most of the interactions that the protein experiences, as the headgroups form the majority of the contacts between the protein and the bilayer.

## Discussion

We have performed simulations of the Dok7 PH domain with complex lipid bilayers containing PIP_2_ and PIP_3_ molecules. Our simulations have shown that PIP molecules were needed for the formation of a stable Dok7 PH/bilayer complex. Furthermore, binding of the PH domain to the bilayer was via interactions of the β1/β2 and β3/β4 loops, i.e. in the region that was previously proposed by Bergamin et al. [13] to be the PIP binding site.

Calculation of density landscapes allowed us to quantify the Dok7 PH domain binding to the bilayer. Our analysis suggests that in the presence of PIP_2_ molecules in the bilayer, the Dok7 PH domain prefers to bind in the *canonical* binding mode, similar to that seen for other PH domains [16,17,39]. When Dok7 bound to bilayers containing PIP_3_, the height and width of the density in the density landscapes was a little smaller compared to that observed for binding to the PIP_2_-containing bilayer. The relatively small difference in binding probabilities correlates with the observation of micromolar dissociation constants for *both* PIP_2_ and PIP_3_ binding to Dok7 [13]. The simulations suggest slightly weaker binding of PIP_3_ than of PIP_2_, whilst the experimental studies suggest slightly stronger binding of PIP_3_. However, there are several differences between the simulations and the experiments respectively, namely: the protein construct (PH domain vs. Dok7); lipids used (PIPs vs. fluorescent derivatives of PIPs); and assay conditions (PIPs in a bilayer vs. in the presence of detergent). The fact that in CG simulations, Dok7 binds to both PIP_2_ and PIP_3_ with little apparent difference between binding probabilities, is encouraging, especially when compared to the fluorescence polarization assays. This suggests that the Dok7 PH domain may bind to both PIP_2_ and PIP_3_. However, *in vivo* PIP_2_ is likely to be the major ligand, given its higher content in plasma membranes.

Recently, a crystal structure of the PH domain of the Arf GAP ASAP1 was solved in complex with dibutyryl-PI(4,5)P_2_ lipids [36]. This provides direct structural evidence for the presence of both a canonical (C) and an atypical (A) PIP-binding site in a single PH domain. This correlates well with the two PIP-binding sites observed in our simulations (see Fig 4 and above). These two binding sites for PIP molecules on Dok7 contribute to the clustering of both PIP_2_ and PIP_3_ observed in our simulations. This clustering of PIP molecules may aid the association of Dok7 with the bilayer and thereby facilitate formation of a complex with MuSK.

In summary, the PH domain of Dok7 associates with the bilayer via a canonical binding mode observed for other PH domains, as evidenced by the two-dimensional density landscapes we have calculated. By virtue of possessing two binding sites for PIPs, Dok7 causes clustering of PIP lipids around it, potentially facilitating its interaction with MuSK enabling downstream signaling transduction. The simulation methodology employed in this work may be readily applied to other peripheral membrane proteins. Alongside methods such as pMD-Membrane [40] which enable identification of possible ligand binding sites on membrane bound proteins, this computational approach may also facilitate discovery of druggable sites on protein/membrane complexes involved in membrane signaling pathways. In the context of Dok7, the characterization of the nature of PH-domain mediated targeting to cell membranes is a key first step in exploiting the nature of the Dok7/MuSK/membrane complex and its role in signaling.

## Methods

### Modelling of Dok7

The published structure of Dok7 (PDB id: 3ML4) was used as a starting point, using Modeller 9v8 [41] to model the missing unstructured regions between strands β1 and β2 in the PH domain.

### CG-MD simulations

CG-MD simulations were performed using the MARTINI2.1 forcefield [42,43] with an elastic network (with a cutoff of 0.7 nm) applied to the protein. The protein was inserted into a box with a preformed bilayer of 356 lipids. The box was then solvated with CG water, and ions were added at physiological concentration (150 mM). Using a high-throughput approach, this was done 20 times, with the protein being placed in the box with a different orientation each time to ensure different initial configurations between the repeat simulations, and to ensure there was no bias in orientation of binding to the bilayer. These simulations were equilibrated then run for 1 μs each.

All CG-MD simulations were run with GROMACS 4.5.5 (www.gromacs.org) using the Berendsen thermostat and barostat for temperature and pressure coupling [44]. The LINCS algorithm was used to constrain bond length [45]. The integrator used was md, group neighbour lists were used, and Shift was used. Each simulation was equilibrated for 2.5 ns, then run using a time step of 20 fs at a temperature of 323 K. For each CG simulation, there was ~15,000 water particles in a box that was approximately 11 nm x 11 nm x 23.5 nm. CG to atomistic conversion was done using a fragment-based approach [46].

### Calculation of density landscapes

To construct these density landscapes, we have merged all 20 simulations for each system. A two-dimensional histogram of *R*_*zz*_ and *d*_*z*_ was then derived from the ensemble of simulations, where *d*_*z*_ is the perpendicular distance between the centre of mass of the Dok7 PH domain and the centre of mass of the bilayer and *R*_*zz*_ is the *zz* component of the rotational matrix required for least squares fitting of an orientation onto a reference orientation. *R*_*zz*_ was calculated by using the *g_rotmat* command in GROMACS. The normalization of the 2-dimensional histogram was done by dividing each bin by the number of repeat simulations (20 for CG) multiplied by the total number of frames in each repeat simulation and by the bin area. After the calculation of the landscapes, the density in each bin was divided by the density in the global minimum to obtain normalized densities.

### Atomistic MD simulations

Representative CG snapshots were chosen which corresponded to the distance and *R*_*zz*_ value of M1 from the coarse-grain simulations. Note that “distance” refers to the distance between the COM of the protein and the COM of the bilayer. These were then used as starting points for atomistic simulations. The AT model of the protein was generated from the CG model as described in Stansfeld *et al*. [46]. All simulations have been performed using the GROMOS96 43a1 force field [47]. The Parinello-Rahman barostat [48] and Berendsen thermostat [44] was used (T = 323 K), with the LINCS algorithm [45] used to constrain bond lengths. Particle Mesh Ewald (PME) [49] was used for long-range (cutoff = 1.2 nm) electrostatics. The simulation in a box size was 10.5 nm x 10.5 nm x 13.5 nm. Systems were equilibrated for 1 ns with the C_α_ atoms restrained (force constant 1000 kJ/mol/nm^2^), followed by 300 ns of unrestrained simulations. Analysis was performed using GROMACS [37], VMD [50], and locally written scripts.

## Supporting Information

S1 Fig Coarse-grained structure of PI(4,5)P_2_ and PI(3,4,5)P_3_ molecules. Images of the coarse-grained models of the PI(4,5)P_2_ **(A)** and PI(3,4,5)P_3_ **(B)** lipids used in the simulations. The headgroups of the lipids are in van der Waals representation, whilst the tails are in licorice representation. PI(4,5)P_2_ is in purple, and PI(3,4,5)P_3_ is in orange.
(TIF)

S2 Fig Convergence analysis for Dok7 PH domain binding to PIP_2_. Density landscapes for the CG-PH-pip2 system when it is separated into 4 x 5 μs, 2 x 10 μs and 1 x 20 μs densities.
(TIF)

S3 Fig Lipid contact analysis between Dok7 and ASAP1. PIP binding sites observed in atomistic simulations of **(A)** Dok7; **(B)** ASAP1 (see [38] for details); and **(C)** a structural alignment of the two PH domains for comparison. Dok7 is in yellow cartoon, ASAP1 is in ochre cartoon, the PIP_2_ that binds to Dok7 is in purple CPK and licorice, and the PIP_2_ that binds to ASAP1 is in magenta CPK and licorice. This is further analysed by calculating averaged normalised lipid contacts for the simulations of **(D)** ASAP1 and **(E)** Dok7. The β1/ β2 and β3/ β4 loops are highlighted on the graphs with red lines, and the *canonical* and *atypical* sites are labelled with a “C” and “A” respectively.
(TIF)

S4 Fig Dok7 PH domain’s interactions with PIP_2_ and PIP_3_ lipids. Number of contacts of Dok7’s PH domain with the headgroups **(A)**, phosphates **(B)**, and tails **(C)** of PIP_2_, and the headgroups **(D)**, phosphates **(E)**, and tails **(F)** of PIP_3_. PC and PS lipids are represented by either a red or green line, respectively; PIP_2_ lipids are represented by a purple line, and PIP_3_ lipids are represented by an orange line.
(TIF)

S5 Fig Secondary structure analysis of Dok7 PH atomistic simulations. **(A)** Secondary structure analysis of all Dok7 PH atomistic simulations. Simulations containing PIP_2_ are in the left column, whilst the simulations containing PIP_3_ are in the right column. Each individual simulation is shown as a separate graph, with the different secondary structures highlighted in blue (α-helix), red (β-sheet), black (coil), green (bend), yellow (loop), orange (β bridge), purple (5-helix), and pink (3-helix). **(B)** Root mean square fluctuation (RMSF) of each residue on Dok7’s PH domain for simulations with PIP_2_ (left) and PIP_3_ (right). Each replicate is shown in a different colour.
(TIF)

S6 Fig Hydrogen bond analysis for Dok7 PH domain. Hydrogen bonds between the Dok7 PH domain with **(A)** PIP_2_ (above) and **(B)** PIP_3_ (below) from the three repeat atomistic simulations (rep1 to 3).
(TIF)

S7 Fig Hydrogen bond analysis for key residues on Dok7 PH domain. Hydrogen bonds of selected residues (K18, K20, R22 and R54) on the Dok7 PH domain with PIP_2_ **(A)** and PIP_3_ **(B)**. The colours correspond to the three repeat atomistic simulations.
(TIF)

S8 Fig Atomistic lipid contacts. Atomistic lipid contacts broken down into headgroup **(A)**, phosphates **(B)**, and tails **(C)** of PIP_2_, and the headgroups **(D)**, phosphates **(E)**, and tails **(F)** of PIP_3_. PC is shown in red, PS in green, PIP_2_ in purple, and PIP_3_ in orange.
(TIF)

S1 File Structure of Dok7 PH domain with the lipid bilayer. PDB file of the Dok7 PH domain with a PC/PS/PIP_2_ bilayer that is aligned with the Arf GAP ASAP1 structure.
(PDB)
