# Supplementary figures and images for: Multiscale Simulations Suggest a Mechanism for the Association of the Dok7 PH Domain with PIP-Containing Membranes
Source: PLoS Comput Biol. 2016 Jul 26;12(7):e1005028. doi: 10.1371/journal.pcbi.1005028 (PMC4961371; doi:10.1371/journal.pcbi.1005028)

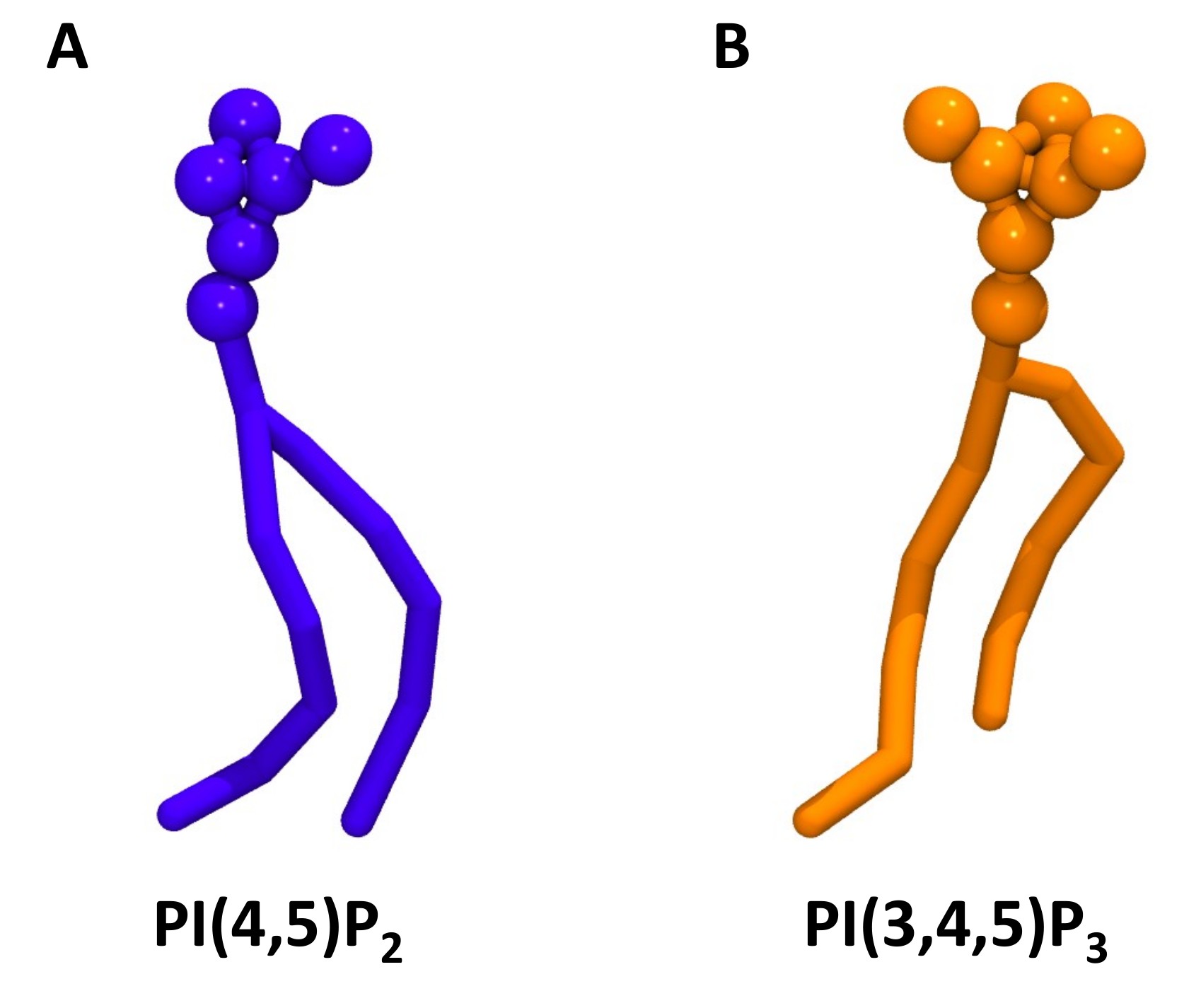

Supplement: S1 Fig — Images of the coarse-grained models of the PI(4,5)P2 (A) and PI(3,4,5)P3 (B) lipids used in the simulations. The headgroups of the lipids are in van der Waals representation, whilst the tails are in licorice representation. PI(4,5)P2 is in purple, and PI(3,4,5)P3 is in orange. (TIF) [file pcbi.1005028.s001.tif]

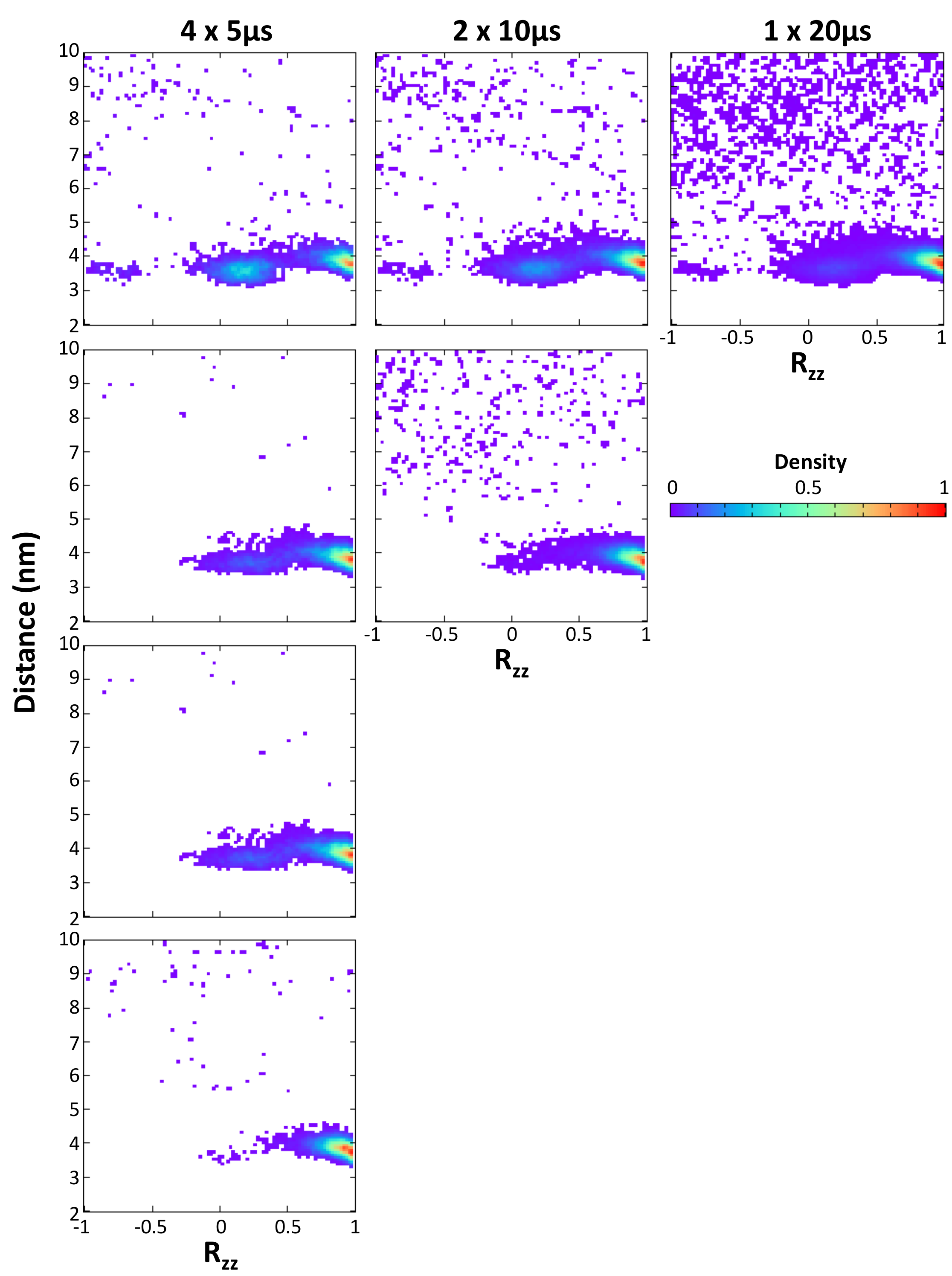

Supplement: S2 Fig — Density landscapes for the CG-PH-pip2 system when it is separated into 4 x 5 μs, 2 x 10 μs and 1 x 20 μs densities. (TIF) [file pcbi.1005028.s002.tif]

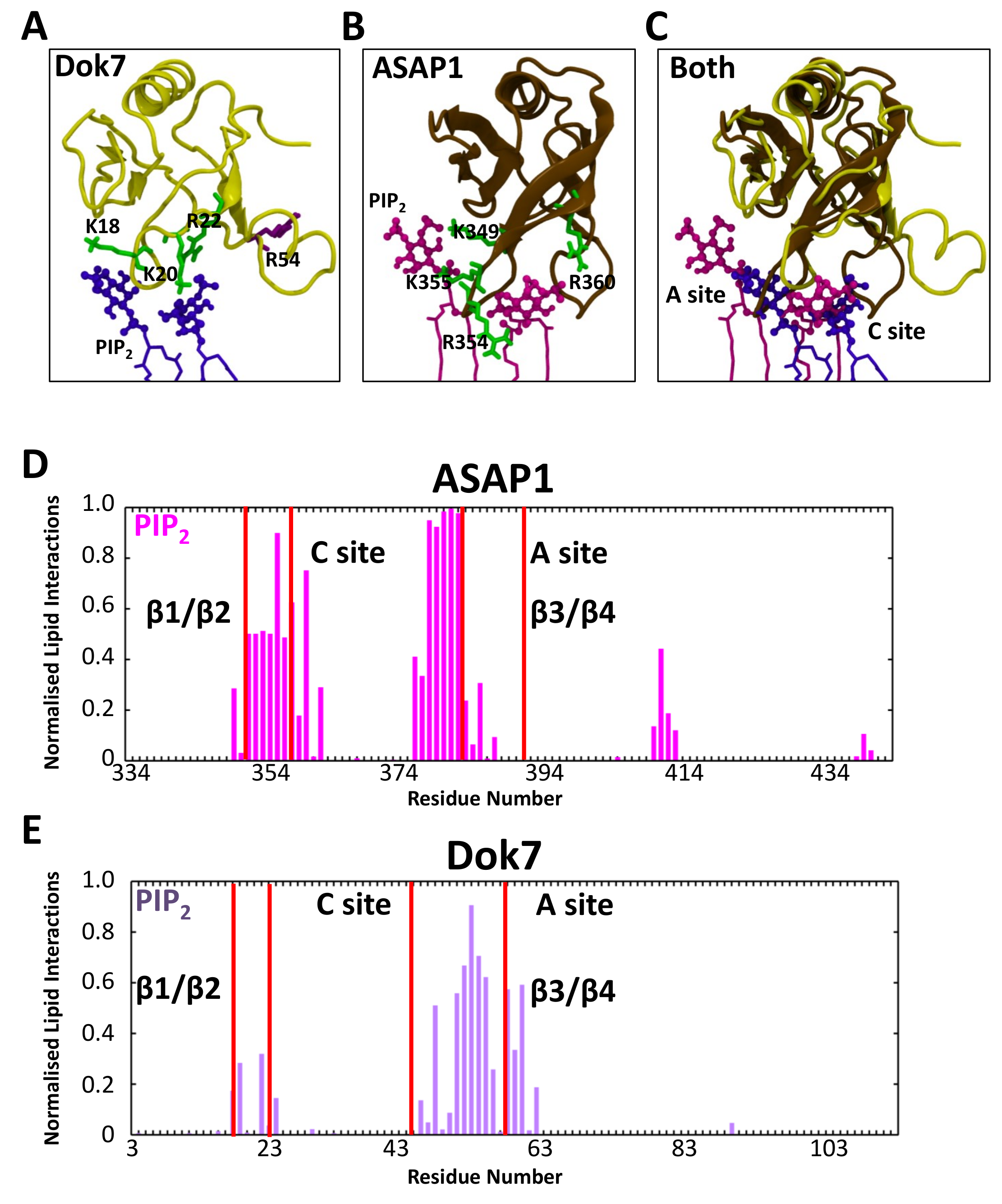

Supplement: S3 Fig — PIP binding sites observed in atomistic simulations of (A) Dok7; (B) ASAP1 (see [38] for details); and (C) a structural alignment of the two PH domains for comparison. Dok7 is in yellow cartoon, ASAP1 is in ochre cartoon, the PIP2 that binds to Dok7 is in purple CPK and licorice, and the PIP2 that binds to ASAP1 is in magenta CPK and licorice. This is further analysed by calculating averaged normalised lipid contacts for the simulations of (D) ASAP1 and (E) Dok7. The β1/ β2 and β3/ β4 loops are highlighted on the graphs with red lines, and the canonical and atypical sites are labelled with a “C” and “A” respectively. (TIF) [file pcbi.1005028.s003.tif]

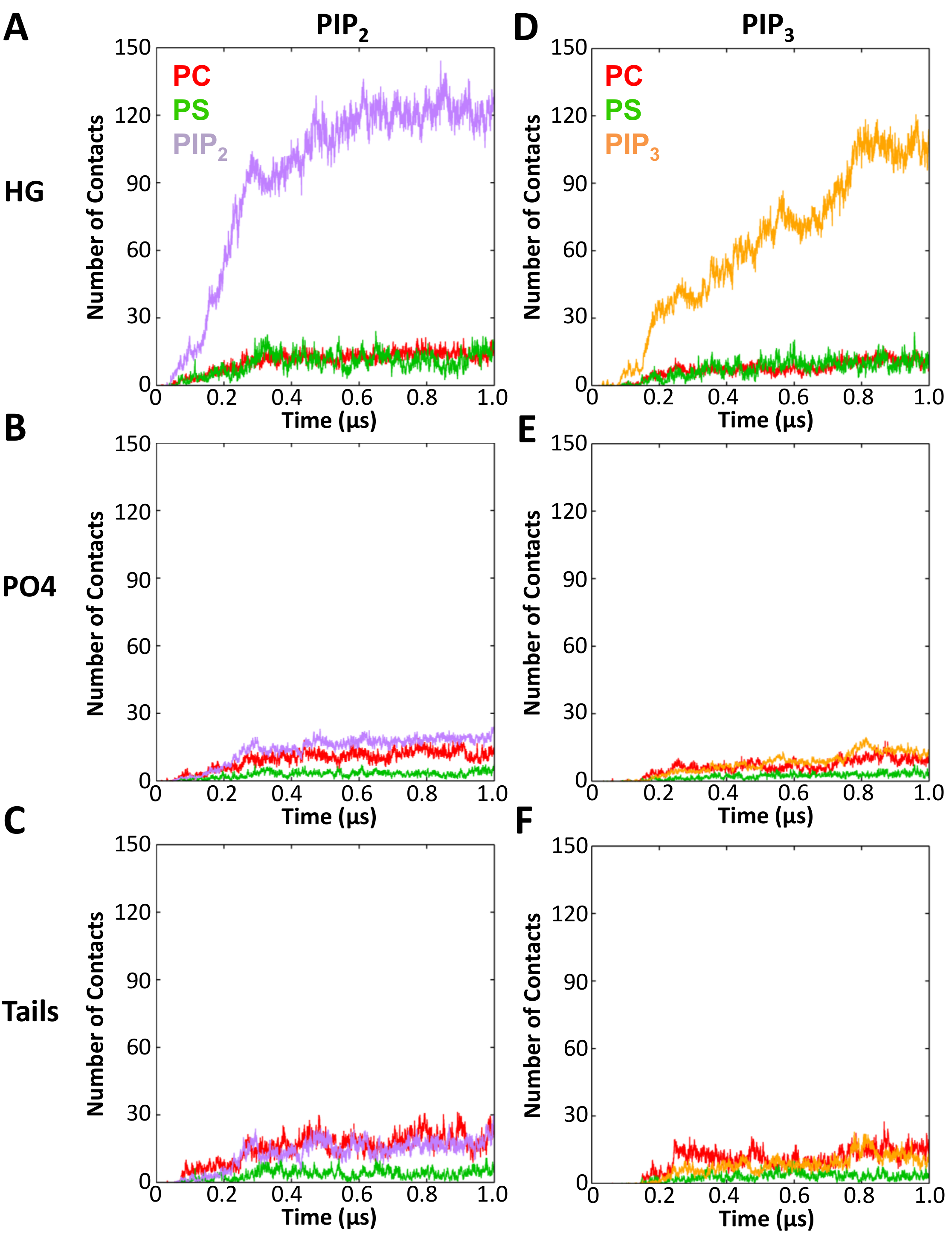

Supplement: S4 Fig — Number of contacts of Dok7’s PH domain with the headgroups (A), phosphates (B), and tails (C) of PIP2, and the headgroups (D), phosphates (E), and tails (F) of PIP3. PC and PS lipids are represented by either a red or green line, respectively; PIP2 lipids are represented by a purple line, and PIP3 lipids are represented by an orange line. (TIF) [file pcbi.1005028.s004.tif]

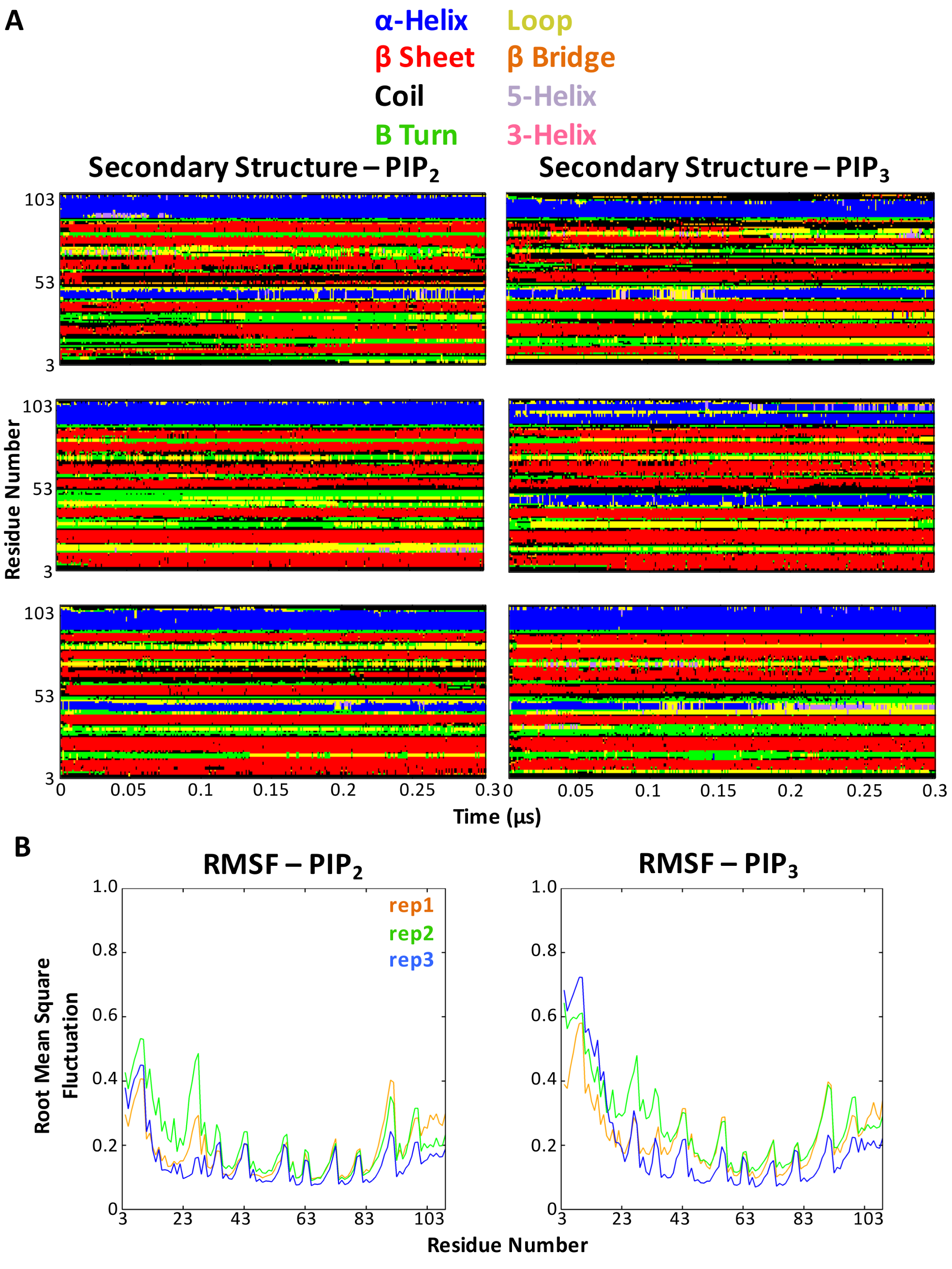

Supplement: S5 Fig — (A) Secondary structure analysis of all Dok7 PH atomistic simulations. Simulations containing PIP2 are in the left column, whilst the simulations containing PIP3 are in the right column. Each individual simulation is shown as a separate graph, with the different secondary structures highlighted in blue (α-helix), red (β-sheet), black (coil), green (bend), yellow (loop), orange (β bridge), purple (5-helix), and pink (3-helix). (B) Root mean square fluctuation (RMSF) of each residue on Dok7’s PH domain for simulations with PIP2 (left) and PIP3 (right). Each replicate is shown in a different colour. (TIF) [file pcbi.1005028.s005.tif]

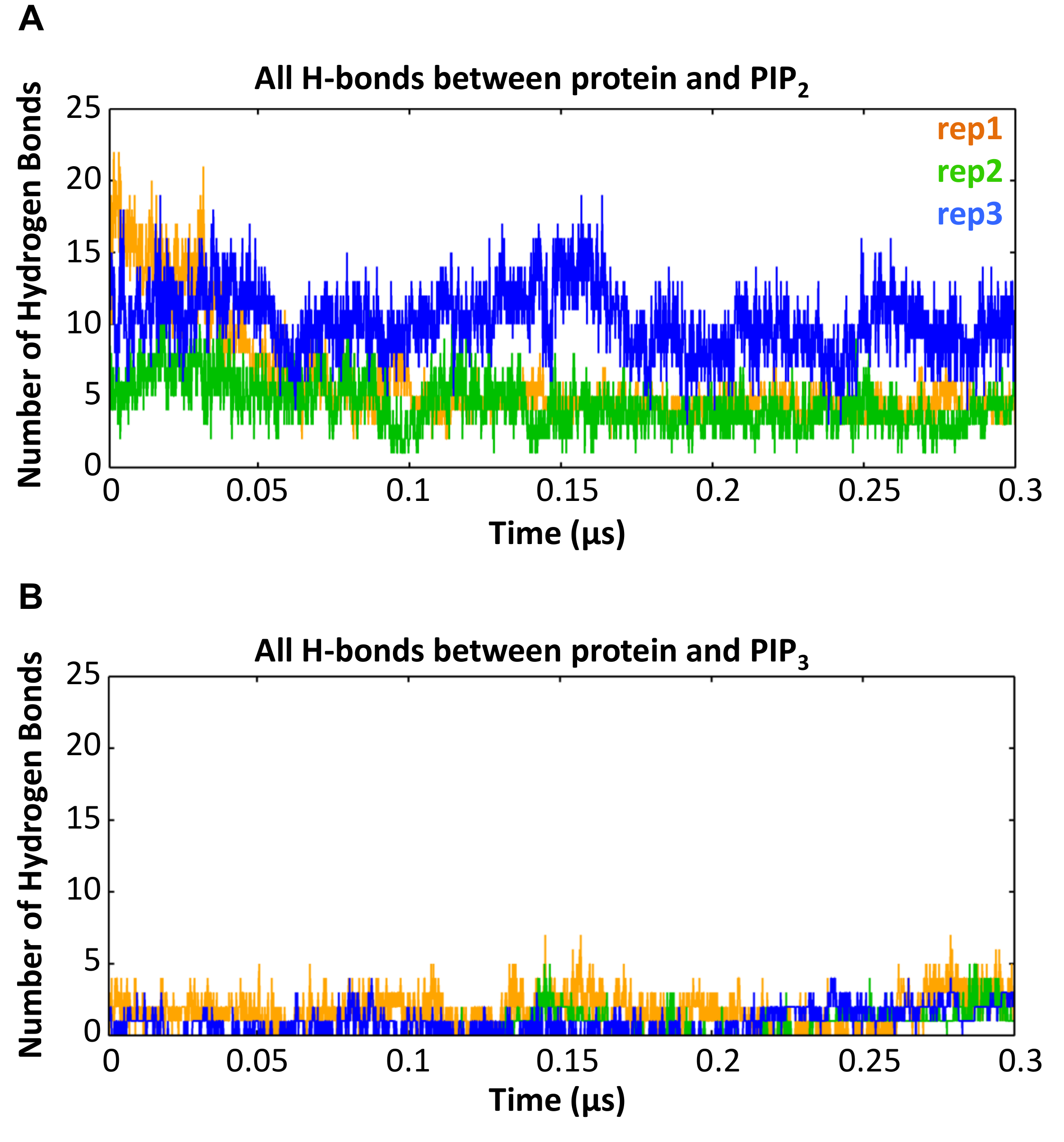

Supplement: S6 Fig — Hydrogen bonds between the Dok7 PH domain with (A) PIP2 (above) and (B) PIP3 (below) from the three repeat atomistic simulations (rep1 to 3). (TIF) [file pcbi.1005028.s006.tif]

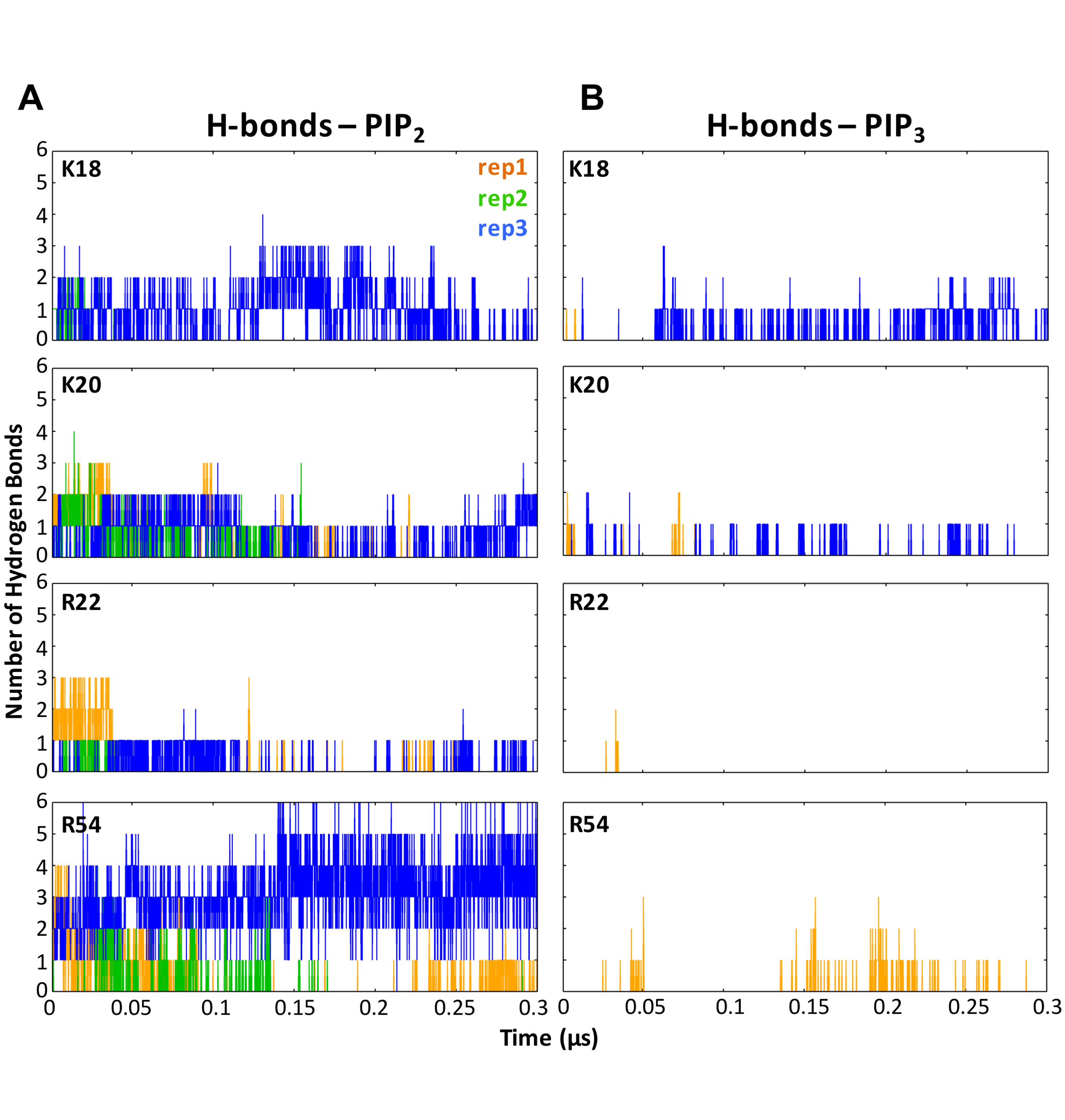

Supplement: S7 Fig — Hydrogen bonds of selected residues (K18, K20, R22 and R54) on the Dok7 PH domain with PIP2 (A) and PIP3 (B). The colours correspond to the three repeat atomistic simulations. (TIF) [file pcbi.1005028.s007.tif]

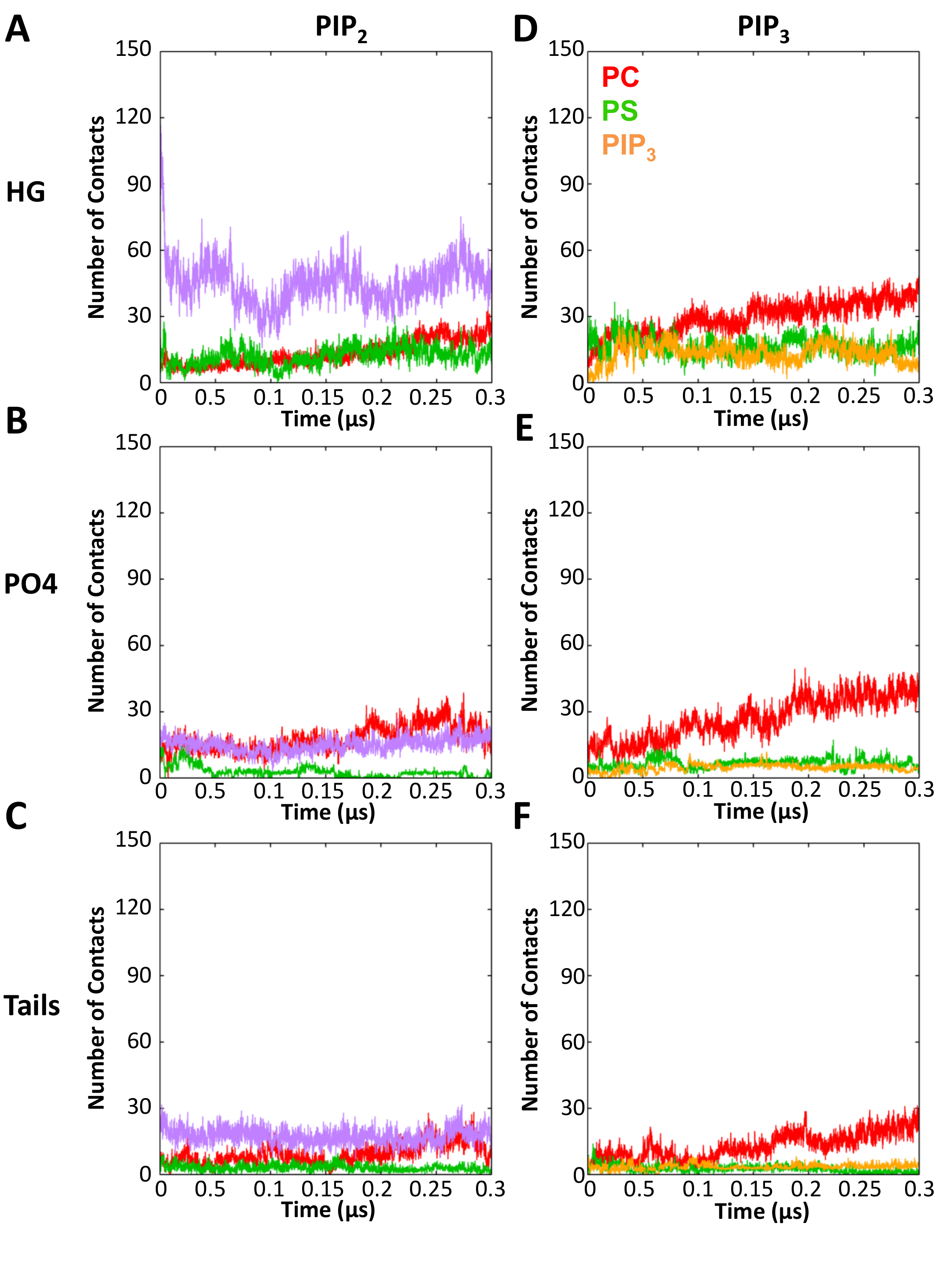

Supplement: S8 Fig — Atomistic lipid contacts broken down into headgroup (A), phosphates (B), and tails (C) of PIP2, and the headgroups (D), phosphates (E), and tails (F) of PIP3. PC is shown in red, PS in green, PIP2 in purple, and PIP3 in orange. (TIF) [file pcbi.1005028.s008.tif]
